# Clinical utility of gene panel-based testing for hereditary myelodysplastic syndrome/acute leukemia predisposition syndromes

**DOI:** 10.1038/leu.2017.28

**Published:** 2017-02-07

**Authors:** L Guidugli, A K Johnson, G Alkorta-Aranburu, V Nelakuditi, K Arndt, J E Churpek, L A Godley, D Townsley, N S Young, C Fitzpatrick, D del Gaudio, S Das, Z Li

**Affiliations:** 1 Department of Human Genetics, The University of Chicago, Chicago, IL, USA; 2 Department of Medicine, Comprehensive Cancer Center and Center for Clinical Cancer Genetics, The University of Chicago, Chicago, IL, USA; 3 Hematology Branch, Cell Biology Section, National Institutes of Health, National Heart, Lung, and Blood Institute, Bethesda, MD, USA; 4 Department of Pathology, The University of Chicago, Chicago, IL, USA

Myelodysplastic syndrome (MDS) and acute leukemia (AL) are clinically diverse and genetically heterogeneous groups of hematological malignancies. Hereditary forms of MDS/AL were considered rare, but have been increasingly recognized in recent years.^1, 2, 3^ Pathogenic variants in a single gene can predispose carriers to an increased lifetime risk of primary MDS and/or AL. Hereditary MDS/AL can occur in the context of familial MDS/AL that have MDS/AL as the principal clinical feature, or arise from inherited bone marrow failure syndromes (IBMFS), such as Fanconi anemia (FA), dyskeratosis congenita/telomerase biology disorders (TBD), Diamond–Blackfan anemia and severe congenital neutropenia.^1, 4^ Within the past decade, nearly a dozen adult-onset familial MDS/AL syndromes have been defined. These include thrombocytopenia with associated myeloid malignancies caused by germ line mutations in *RUNX1*,*ANKRD26* and *ETV6*;*GATA2*-associated syndromes (Emberger syndrome; MonoMAC syndrome; immunodeficiency); familial MDS and acute myeloid leukemia caused by mutations in *CEBPA*, *DDX41* and*SRP72*; and TBD due to mutations in *TERT* or *TERC.*^2^ Although the majority of patients with classic IBMFS are diagnosed in childhood, some patients have no or only subtle extra hematopoietic manifestations and may present in adulthood with MDS or AL.^2, 5^

A few studies have shown that genetic abnormalities exist in 11–37% of families with hereditary MDS/AL.^6, 7, 8, 9, 10^ The recognition of patients with a hereditary predisposition to MDS/AL is particularly important for hematopoietic stem cell transplantation donor selection, pre-transplant planning and post-transplant care.^11^ The correct clinical diagnosis is also important to avoid the risk of life-threatening toxicities with inappropriate therapy, for long-term cancer surveillance and prognosis, and for identification of at-risk or affected family members.^5^ Clinical guidelines for the care of MDS/AL predispositions are now emerging.^1, 2, 3^ To reflect the increasing recognition and clinical awareness of hereditary hematological malignancies, the World Health Organization (WHO) has included germ line predisposition to myeloid malignancies in the forthcoming WHO classification guidelines.^12^ However, the application of genetic testing on hereditary MDS/AL in clinical practice has never been systematically reported.

Given the phenotypic overlap of the known hereditary MDS/AL predisposition syndromes, a gene panel-based approach to genetic testing is preferred, as it offers the ability to analyze multiple genes simultaneously and cost-effectively. Our College of American Pathologists certified and Clinical Laboratory Improvement Amendments-licensed laboratory is the first to provide comprehensive clinical testing via a combination of multiple next-generation sequencing and array comparative genomic hybridization-based panel tests to evaluate genetic predisposition to MDS/AL. Multiple gene panels are available, including a familial MDS/AL panel, IBMFS panel, and panels for FA, dyskeratosis congenita/TBD, Diamond–Blackfan anemia and severe congenital neutropenia (Table 1 and Supplementary Table 1). Cultured skin fibroblasts are the preferred tissue for germ line mutation testing in patients with hematological malignancy as they provide higher quality and quantity of DNA compared to hair roots and nail clippings. The targeted next-generation sequencing was performed using Illumina technology (San Diego, CA, USA). The high-density exon-targeted array comparative genomic hybridization is custom designed using Agilent Technology (Santa Clara, CA, USA). The variant interpretation follows the standards and guidelines for the interpretation of sequence variants from the American College of Medical Genetics and Genomics.^13^

A total of 197 patients (110 females and 87 males) were referred to our laboratory for MDS/AL predisposition gene panel testing from October 2014 to June 2016. The patient age at the time of testing ranged from 1 to 84 years in 65 children and 132 adults. Seventy-eight patients were referred for testing for the familial MDS/AL panel, 86 for the IBMFS panel, 15 for the dyskeratosis congenita/TBD panel and 12 for multiple panel testing. In addition, a total of six patients were referred for specific testing of FA, Diamond–Blackfan anemia and severe congenital neutropenia (Table 1).

The overall molecular diagnostic rate was 19% (37 of 197) with 15% in children and 21% in adults (Table 1). Pathogenic/likely pathogenic variants were identified in 14 (18%) patients tested on the familial MDS/AL panel, 13 (16%) patients tested on the IBMFS panel, 5 (33%) patients tested on the dyskeratosis congenita/TBD panel, 1 (33%) patients tested on the FA panel and 3 (25%) patients tested on multiple panels. The most frequently affected genes were *FANCA* (four of the five cases from the IBMFS panel) and *GATA2* (four of the five cases from the familial MDS/AL panel) (Table 2, Supplementary Figures 1 and 2). Unexpectedly, three of the five patients with *FANCA* deleterious variants were adults, and presented with aplastic anemia or acute myeloid leukemia as the major phenotype. Patient 5 was a child without typical features of FA at the time of the testing. Only Patient 2 was a child diagnosed clinically with FA. Diepoxybutane testing was performed on Patients 2 and 4, and both were abnormal. In these cases, genetic testing informed the clinical diagnosis in the absence of typical FA features. Our result is also consistent with previous reports that *GATA2* is one of the more commonly mutated genes in MDS/AL predisposition syndromes.^8, 14^ Four of the five *GATA2* deleterious variants were novel, and four of them were located in the ZF2 domain, further emphasizing this domain as a mutational hot spot (Table 2 and Supplementary Figure 2). *TERT*, *DDX41* and *RUNX1* were the next frequently mutated genes (Table 2 and Supplementary Figure 2). Overall, 21 novel pathogenic/likely pathogenic variants have been identified (Table 2).

Gene panel testing can aid in the clinical diagnosis of hereditary MDS/AL, particularly in the presence of phenotypic overlap and genetic heterogeneity. For instance, Patient 4, a 19-year-old male with a history of longstanding unexplained thrombocytopenia and learning disability, developed acute myeloid leukemia and was suspected to carry a pathogenic variant in *RUNX1*,*ETV6* or *ANKRD26* (Table 2). However, testing detected two pathogenic variants in *FANCA*, c.2398G>T (p.Glu800*) and c.2601+1G>T (p.?) (Table 2), supporting a diagnosis of FA. Subsequent diepoxybutane testing confirmed the diagnosis of FA and prompted changes in the medical management to avoid substantial morbidity due to intensive chemotherapy.^15^

Our testing has also demonstrated that the identification of pathogenic variants predisposing to MDS/AL has a significant impact on the choice of pre-transplant conditioning and selection of sibling donors. A pathogenic variant in *GATA2* was identified in Patient 8 who was being evaluated for hematopoietic stem cell transplantation at the time of testing, with a sister as a potential donor. The molecular diagnosis in this patient urged follow-up testing to be performed on the sister to prevent potentially devastating consequences associated with the use of a donor who carries the same pathogenic *GATA2* variant.

A total of 106 variants of uncertain significance were identified in 72 (37%) patients (Supplementary Figure 3). As the majority of the genes in these panels are relatively novel, less is known about unique variants seen for the first time. Population genetic data, segregation studies in family members and functional studies may help clarify the nature of some of these variants and reduce the yield of variants of uncertain significance on similar panels. Among the variants of uncertain significance, two were predicted to affect RNA splicing by *in silico* prediction tools (Alamut Visual, Rouen, France), and RNA splicing assays were performed to clarify the nature of these variants. The first, a novel heterozygous *FANCA* variant, c.826+5_826+9del (p.?), in Patient 4, was predicted to affect the canonical splice donor site of exon 9 (Table 2, Supplementary Table 2, Supplementary Figure 4A). RNA splicing assay revealed the presence of an aberrant isoform with a deletion of exon 9 that resulted in a premature stop codon in exon 10 (Supplementary Figures 4B–D). Further review of the next-generation sequencing data revealed an additional large heterozygous deletion of exons 21–28 in *FANCA* in this patient, which was confirmed by array comparative genomic hybridization (Supplementary Figure 4E) and determined to be *in trans* with c.826+5_826+9del. The second, a novel *GATA2* variant, c.857C>T (p.Ala286Val), in Patient 6, was predicted to generate a cryptic splice donor site (Table 2 and Supplementary Table 2). We demonstrated an aberrant isoform with a deletion of 16 bp in exon 3 resulting in a premature stop codon in exon 4 of *GATA2* by RNA splicing analysis of skin fibroblasts with or without cycloheximide treatment (a nonsense-mediated messenger RNA decay inhibitor; Supplementary Figures 5A and B). The results provided sufficient evidence that the c.857C>T is a likely pathogenic variant.

Eighty-seven (44%) patients had negative testing results. Genetic abnormalities in a considerable proportion of patients with a history of MDS/AL predisposition therefore remain uncharacterized, suggesting that additional germ line genetic aberrations exist and remain to be identified. Research studies and periodic follow-up will help establish the genetic basis of the disorders in these patients. Clinical genetic testing also needs to be continually updated with the rapidly growing recognition of additional MDS/AL risk genes and syndromes.

In conclusion, our study demonstrates the utility of genetic testing for hereditary MDS/AL predisposition syndromes. This study has provided a better understanding of the genetic etiology of hereditary MDS/AL predisposition syndromes and broadened the gene mutation spectrum. RNA splicing analysis played an important role in clarifying variant pathogenicity. The cost of these panels is similar to gene panel testing for other genetic disorders. Our gene panel-based testing for the diagnosis of hereditary MDS/AL syndromes is being integrated into clinical hematological malignancy evaluation and the clinical decision-making for personalized treatment considerations.

## Figures and Tables

**Table 1:** Molecular diagnoses in patients tested shown by age of onset (childhood onset, 0–16 years old; adulthood onset, 17 years old and older) and all together

*Panels*	*Patients tested*			*Mutation detected*			*Rate of molecular diagnosis*		
	*Children*	*Adults*	*Total*	*Children*	*Adults*	*Total*	*Children*	*Adults*	*Total*
Familial MDS/AL^a^	18	60	78	4	10	14	22%	17%	18%
IBMFS	28	58	86	1	13	14	4%	22%	16%
DC/TBD	7	8	15	2	3	5	29%	38%	33%
FA	1	2	3	1	NA	1	100%	NA	33%
DBA	1	NA	1	1	NA	1	100%	NA	100%
SCN	2	NA	2	NA	NA	NA	NA	NA	NA
Multiple panels^b^	8	4	12	1	2	3	13%	25%	25%
Total	65	132	197	10	28	38	15%	21%	19%

Abbreviations: AML, acute myeloid leukemia; DBA, Diamond–Blackfan anemia; DC/TBD, dyskeratosis congenita/telomere biology disorders; FA, Fanconi anemia; IBMFS, inherited bone marrow failure syndrome; MDS/AL, myelodysplastic syndrome/acute leukemia; NA, not applicable; SCN, severe congenital neutropenia.

a For patients referred for the familial MDS/AL panel, 36 were from the University of Chicago Medical Center and had documented pathologic confirmation; others were from outside hospitals and most of them were diagnosed with MDS or AML at the time of testing.

b The patient was tested for more than one panel.

**Table 2:** Summary of pathogenic and likely pathogenic variants identified in a total of 38 patients

*Patient*	*Gene*	*DNA*	*Protein*	*Zygosity*	*Inheritance*	*Panel*	*Previously reported (PMID, ClinVar, ExAc) or novel*
1	FANCA	c.1A>G	p.Met1?	Het	AR	IBMF	10090479
		c.3624C>T	p.(=)	Het	AR	IBMF	16084127; 17924555; 22778927
2	FANCA	c.826+5_826+9del	p.?	Het	AR	FA	Novel
		Del exons 21–28	p.?	Het	AR	FA	24584348
3	FANCA	c.1115_1118del	p.Val372Alafs42^a^	Het	AR	IBMF	In ClinVar
		Del exons 15–17	p.?	Het	AR	FA	10521298
4	FANCA	c.2398G>T	p.Glu800^a^	Het	AR	IBMF	Novel
		c.2601+1G>T	p.?	Het	AR	IBMF	Novel
5	FANCA	Del exons 18–43	p.?	Het	AR	Familial MDS/AL	Novel
		c.3482C>T	p.Thr1161Met	Hom	AR	IBMF	0.005% in ExAC
6	GATA2	c.857C>T	p.Ala286Val	Het	AD	Familial MDS/AL	Novel
7	GATA2	c.1054T>C	p.Cys352Arg	Het	AD	Familial MDS/AL	Novel
8	GATA2	c.1081C>G	p.Arg361Gly	Het	AD	Familial MDS/AL	Novel
9	GATA2	c.1084C>T	p.Arg362^a^	Het	AD	IBMF	Novel
10	GATA2	c.1192C>T	p.Arg398Trp	Het	AD	Familial MDS/AL	21670465; 25111582; 24345756; 26214525; 25359990
11	TERT	c.604G>A	p.Ala202Thr	Het	AR/AD	IBMF	15814878
12	TERT	c.1620C>G	p.Ile540Met	Hom	AD/AR	DC/TBD	Novel
13	TERT	c.2146G>A	p.Ala716Thr	Het	AR/AD	IBMF	Novel
14	TERT	c.3150G>C	p.Lys1050Asn	Het	AD/AR	Familial MDS/AL	26024875
15	DDX41	c.3G>A	p.Met1?	Het	AD	Familial MDS/AL	26712909; 0.005% in ExAC
16	DDX41	c.323del	p.Lys108Serfs3^a^	Het	AD	Familial MDS/AL	Novel
17	DDX41	c.1016G>T	p.Arg339Leu	Het	AD	Familial MDS/AL	Novel
18	RUNX1	Del exons 1–2	p.?	Het	AD	IBMF	Novel
19	RUNX1	c.352-1G>A	p.?	Het	AD	Familial MDS/AL	Novel
20	RUNX1	c.557T>A	p.Val186Asp	Het	AD	IBMF	Novel
21	RTEL1	c.3028C>T	p.Arg1010^a^	Het	AR/AD	DC/TBD	23329068; 0.008% in ExAC
22	RTEL1	c.3791G>A	p.Arg1264His	Het	AR/AD	DC/TBD	25607374; 0.008% in ExAC
23	SBDS	c.258+2T>C	p.?	Het	AR/AD	IBMF	12496757; 15284109; 15942154; 17478638; 0.395% in ExAC
24	SBDS	c.258+2T>C	p.?	Het	AR/AD	IBMF	12496757; 15284109; 15942154; 17478638; 0.395% ExAC
25	ANKRD26	c.-119C>G	p.?	Het	AD	Familial MDS/AL	Novel
26	ETV6	c.614del	p.Leu205Argfs4^a^	Het	AD	Familial MDS/AL	Novel
27	TP53	c.869G>A	p.Arg290His	Het	AD	Familial MDS/AL	10435620; 17541742; 22811390; 19468865; 26086041; 25925845; 0.016% in ExAC
28	CEBPA	c.119dup	p.Gln41Alafs67^a^	Het	AD	Familial MDS/AL	Novel
29	CHEK2	c.1283C>T	p.Ser428Phe	Het	AD	Familial MDS/AL	15649950; 18085035; 0.031% in ExAC
30	TINF2	c.845G>A	p.Arg282His	Het	AD	DC/TBD	18979121; 18252230; 18669893
31	RPS26	c.55C>T	p.Gln19^a^	Het	AD	DBA	Novel
32	FANCL	c.1007_1009del	p.Ala291Val	Het	AR	IBMF	0.026% in ExAC
33	MPL	c.972del	p.Arg325Glufs44^a^	Het	AR/AD	IBMF	0.001% in ExAC
34	CTC1	c.2954_2956del	p.Cys985del	Hom	AR	IBMF	0.009% in ExAC
35	RPS19	c.356+3A>C	p.?	Het	AD	IBMF	Novel
36	BRIP1	c.139C>G	p.Pro47Ala	Het	AR/AD	IBMF	11301010; 14983014
36	TERC	n.287C>G	NA	Het	AD	IBMF	21931702
37^a^	G6PC3	c.130C>T	p.Pro44Ser	Het	AR	IBMF	23298686; 22469094; 21264919; 0.005% in ExAC
38	DKC1	c.1255T>A	p.Tyr419Asn	Hem	X-linked	DC/TBD	Novel

Abbreviations: AD, autosomal domiant; AR, autosomal recessive; DBA, Diamond–Blackfan anemia; DC/TBD, dyskeratosis congenita/telomere biology disorders; ExAC, Exome Aggregation Consortium; FA, Fanconi anemia; Hem, hemizygote; Het, heterozygote; Hom, homozygote; IBMF, inherited bone marrow failure; MDS/AL, myelodysplastic syndrome/acute leukemia.

a Only one pathogenic variant detected in *G6PC3*. It cannot be ruled out that a second pathogenic variant in the *G6PC3* is present that could not be detected by the assay.
