# Supplementary material for: Clinical utility of gene panel-based testing for hereditary myelodysplastic syndrome/acute leukemia predisposition syndromes
Source: Leukemia. 2017 Feb 7;31(5):1226–9. doi: 10.1038/leu.2017.28 (PMC5420790; doi:10.1038/leu.2017.28)
Supplement: Supplementary Information [file leu201728x1.docx]

**Supplemental Information**

**Clinical Utility of Gene Panel-based Testing for Hereditary Myelodysplastic Syndrome/Acute Leukemia Predisposition Syndromes**

Lucia Guidugli^1^, Amy Knight Johnson^1^, Gorka Alkorta-Aranburu^1^, Viswateja Nelakuditi^1^, Kelly Arndt^1^, Jane E. Churpek^2^, Lucy A. Godley^2^, Danielle Townsley^3^, Neal S Young^3^, Carrie Fitzpatrick^4^, Daniela del Gaudio^1^, Soma Das^1^, Zejuan Li^1*^

^1^Department of Human Genetics, The University of Chicago, Chicago, IL 60637.

^2^Department of Medicine, Comprehensive Cancer Center and Center for Clinical Cancer Genetics, The University of Chicago, Chicago, IL 60637.

^3^Hematology Branch, Cell Biology Section, National Institutes of Health, National Heart, Lung, and Blood Institute, Bethesda, MD 20814.

^4^Department of Pathology, the University of Chicago, Chicago, IL 60637.

^*^ Corresponding author

**Materials and Methods**

**Clinical hereditary MDS/AL predisposition gene panel design**

The panels were designed based on the most common clinical presentations. Familial MDS/AL panel was designed for patients with younger at presentation than individuals with sporadic disease and/or with an unusual family history of MDS/AL. Genes associated with adult-onset familial MDS/AL syndromes such as *RUNX1, ANKRD26,* *ETV6*; *GATA2*, *CEBPA*, *DDX41,* *SRP72* among others were selected for the Familial MDS/AL panel. IBMFS panel was designed for patients with bone marrow failure syndrome phenotype and included genes associated with IBMFS, such as FA, DC/TBD, DBA, and SCN. The panels of FA, DC/TBD, DBA, and SCN were designed for patients with clear diagnosis or highly suspect of a specific disease. The study was approved by the institutional review board. Consent for the research study was provided by the patients or the patients’ parent/guardian.

**Targeted sequencing and data analysis**

Briefly, the custom enrichment design targets the coding regions and flanking intronic regions for all the selected genes. The target regions also include the 5'UTRs of *ANKRD26*, *DKC1*, *TERC* and *TERT,* two intronic regions (c.3724+78 and c.3724+139) in *RTEL1* (NM_032957.4) and one intronic region (c.1017+572) in *GATA2* (NM_032638.4). SureSelect^XT^ Target Enrichment System (Agilent Technologies, Santa Clara, CA) was used for the capture of the genomic regions. Captured DNA library was normalized, pooled, and sequenced using paired-end 150bp reads with an 8 base pair sample-specific index on Illumina NextSeq 500 (Illumina, Inc. San Diego, CA).

The data were analyzed using a custom bioinformatics pipeline developed from publicly available bioinformatics tools. Briefly, the quality and integrity of the sequence data were assessed using FastQC, a Java-based software package that analyses signal purity over the read length and the over-enrichment of particular sequence of the data. The raw data were aligned to the reference human genome using BWA. Next, reads in certain problematic areas were identified and realigned using GATK. Then, the depth and completeness of the data set, defined as alignment statistics, were generated using GATK. On average 800 sequence reads are generated for each base. Variants were called on the final alignment file using GATK’s UnifiedGenotyper tool. The target region for variant analysis is the coding regions of the selected genes, plus 10bp of flanking intronic sequence and specific regions described above. These raw variants were further filtered using GATK’s best practice guidelines to retain high quality variants which were annotated using the Alamut–Batch software and further filtered based on the global population frequency in 1000 Genomes project (1000G) and Exome Sequencing Project (ESP). The final list of variants with minor allele frequency lower than 1% was deposited into a MySQL database for further analysis.

Variants in the selected panels were analyzed. Variant interpretation followed the standards and guidelines for the interpretation of sequence variants from American College of Medical Genetics and Genomics (ACMG). Variants with an allele frequency <1% in the 1000 Genomes Project, ESP and Exome Aggregation Consortium (ExAC) databases were reviewed in detail. Previously reported pathogenic variants were identified using ClinVar, Human Gene Mutation Database (HGMD), the Online Mendelian Inheritance in Man (OMIM), and in locus specific databases such as the Leiden Open Source Variation Database (LOVD) and literature search. PhastCons and phylop were used for conservation analysis. In silico tools such as SIFT, PolyPhen-2, Align-GVGD and MutationTaster and CADD score were used for functional effect prediction. Splicing algorithms, such as SpliceSiteFinder-like, MaxEntScan, NNSPLICE, GeneSplicer and Human Splicing Finder were also utilized to evaluate a potential impact of the variants on the splicing mechanism. All pathogenic and likely pathogenic variants were confirmed by Sanger sequencing.

**Array CGH analysis**

High-density exon-targeted array CGH was developed to detect intragenic deletions/duplications for genes associated with hereditary MDS/AL predisposition syndromes. Intragenic deletions and duplications were analyzed by array CGH for the genes besides *DDX41*, *ETV6* and *UBE2T* on all the panels except for the IBMFS panel. The oligonucleotide probes were designed to target the exonic regions and plus/minus 2 kb from the exon/intron junctions of all the selected genes with an average spacing of 1 probe per ~150 bp in the regions of interest using the SureDesign tool from Agilent Technologies. The array CGH compared DNA content from two differentially labeled genomes. The two genomes, a test (or patient) and a reference (or control), were co-hybridized onto the microarray chip on which the oligonucleotide probes had been immobilized. The signal intensity ratios of the test sample versus the reference sample were then calculated for each probe across the entire genome. The Imagene 9.0 and the Nexus Copy Number 7 software (Biodiscovery, Inc. El Segundo, CA) were used for microarray analysis and for copy number aberration detection. The average minimum detection of copy number variation size is about 400bp. Only the coding and immediate flanking regions of the included genes were analyzed. MLPA and quantitative Real Time PCR analysis were sometimes conducted to confirm findings. All patients tested on the familial MDS/AL, SCN, and DBA panels had both NGS and array CGH testing performed. Array CGH was not performed in 1 patient tested on FA panel and 8 patients on DC/TBD panel because it was not selected by the ordering providers. Patients tested on IBMFS panel did not have array CGH analyzed because array CGH for the IBMFS panel was not available at the time of this study.

**Skin fibroblast cultures**

Fibroblast cultures were initiated from skin punches.  Using sterile technique, fat was removed and the remaining dermal and epidermal layers were manually minced with a scalpel in type I collagenase/αMEM.  The specimen was incubated overnight in collagenase at 37 °C with 5% CO_2_.  To further dissociate cells, the tissue was aspirated through a 20-gauge needle and cultured in AmnioMax medium (Thermo Fisher Scientific, Waltham, MA) in a T25 vented flask.  When cultures reached confluence, cells were trypsinized, washed in HBSS and submitted for DNA extraction.  The success rate of culture was approximately 94%.

**DNA isolation**

Genomic DNA was isolated from skin fibroblasts or blood leukocytes on the AutoGenFlex STAR robotic workstation (Autogen, Inc. Holliston, MA) or using the MagNA Pure Compact DNA isolation system (Roche Applied Science, Inc. Indianapolis, IN) following the manufacturer's instructions.

**RNA extraction and RNA splicing analysis**

Total RNA was isolated from peripheral blood of Patient 4 using the PAXgene PreAnalytiX kit (Qiagen, Germantown, MD) and from skin fibroblasts of Patient 6 using the miRNeasy kit (Qiagen). One step RT-PCR was performed using the OneStep RT-PCR kit (Qiagen). The PCR products were analyzed using the LabChip GX automated Electrophoresis system (PerkinElmer, Inc. Waltham, MA). RT-PCR products were also sequenced by Sanger sequencing.

**
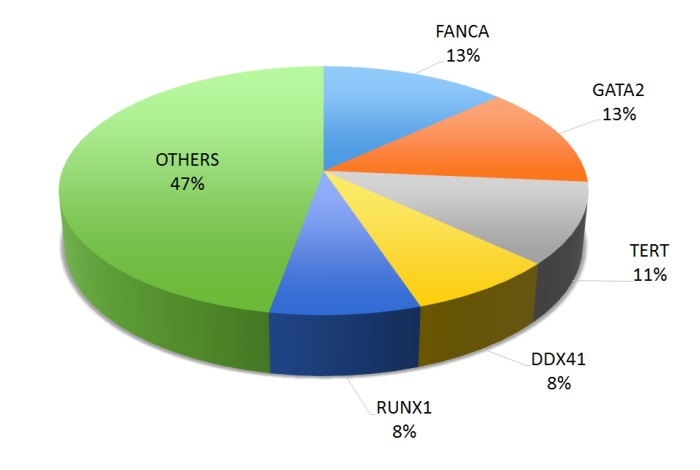
**

**Supplementary Figure 1**: Distribution of pathogenic and likely pathogenic variants identified in 38 patients. Deleterious variants were identified in *FANCA* in 5 patients (13%), in *GATA2* in 5 patients (13%), in *TERT* in 4 patients (11%), in *DDX41* in 3 patients (8%), in *RUNX1* in 3 patients (8%), and in 17 other genes in18 additional patients (47%).

**
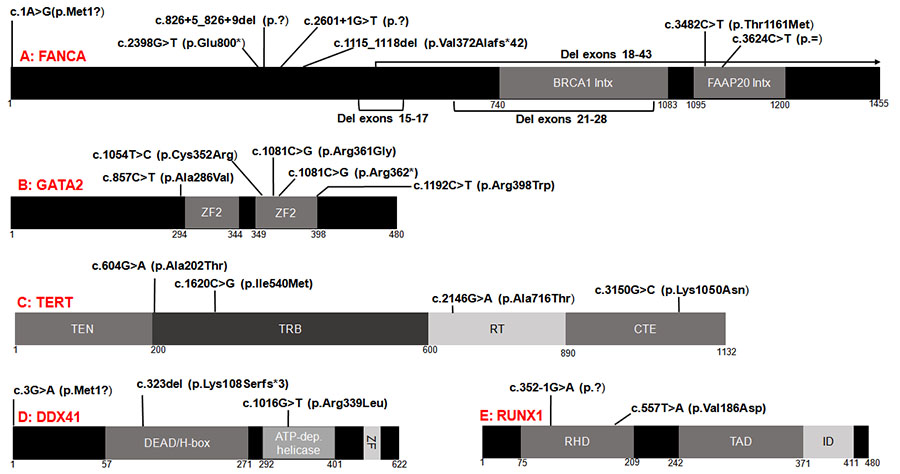
**

**Supplementary Figure 2**: Location of pathogenic and likely pathogenic variants identified in this study and protein functional domains ^1-4^ of FANCA, GATA2, TERT, DDX41 and RUNX1. The first and the last residue are shown for each protein together with the residues that mark the boundaries of the functional domains. (A) FANCA (RefSeq: NM_000135.2; NP_000126.2); BRCA1 Intx corresponds to the domain of interaction with BRCA1^5^, and FAAP20 Intx corresponds to the domain interaction with the protein FAAP20 ^6^. (B) GATA2 (RefSeq: NM_032638.4; NP_116027.2); ZNF1 and ZNF2 correspond to the Zinc finger DNA binding domains that bind specifically to DNA consensus sequence [AT]GATA[AG] promoter elements^7^. (C) TERT (RefSeq: NM_198253.2; NP_937983.2); the TEN domain corresponds to the telomerase essential N-terminal domain, the TRB is the TERT RNA binding domain, the RT is the reverse transcriptase domain and the CTE is the C-terminal extension domain^8^; (D) DDX41 (RefSeq: NM_016222.2; NP_057306.2); the DEAD/H-box is a conserved motif Asp-Glu-Ala-Asp (DEAD), identified in proteins that act as RNA helicases, the ATP-dep. helicase is C-terminal ATP-binding site, and the ZF is a Zinc Finger domain^9^. (E) RUNX1 (RefSeq: NM_001754.4; NP_001745.2); the Runt domain is the region of binding to DNA and of interaction with CBFβ; TD is the transactivation domain, and ID corresponds to the transcription inhibition domain^10^.

**
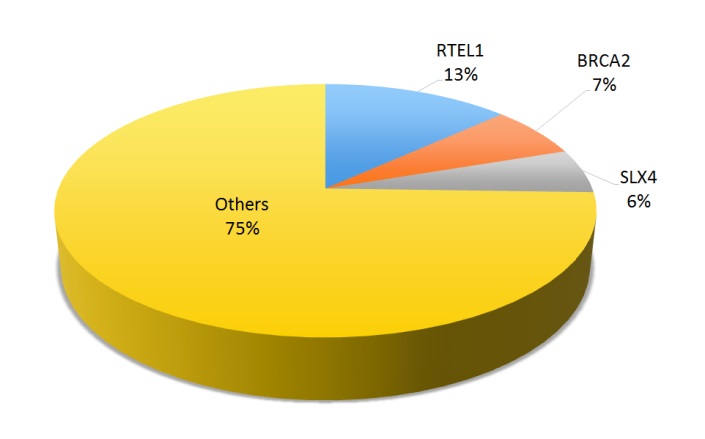
**

**Supplementary Figure 3**: Distribution of VUS identified by the gene panel testing. One hundred and six VUS were identified in 72 patients. Fourteen VUS were identified in *RETL1* (13%), 7 in *BRCA2* (6%), 6 (6%) in *SLX4*, and 79 (75%) in 43 other genes.

**
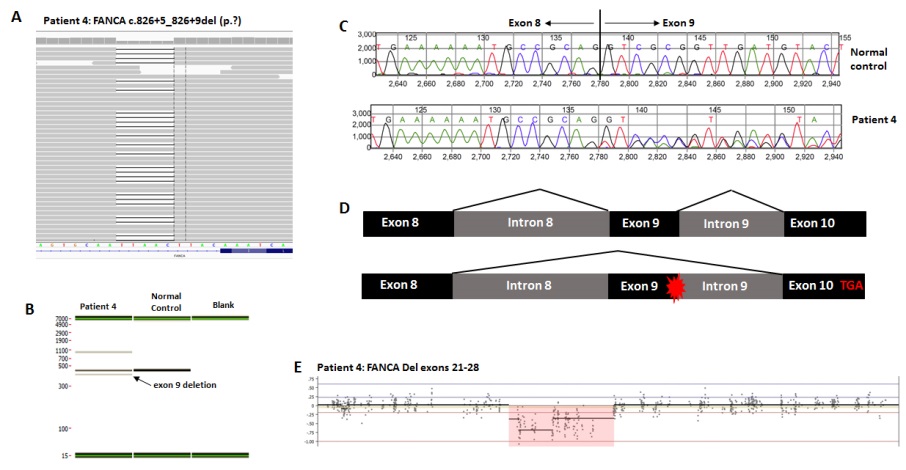
**

**Supplementary Figure 4**: Molecular characterization of two compound heterozygous mutations in *FANCA* in the patient 4. **A**. A heterozygous intronic VUS in *FANCA*, c.826+5_826+9del, was identified by the Fanconi anemia-NGS panel analysis in the patient 4. The variant is visualized with the Integrative Genomics Viewer (IGV). **B**. The *FANCA*-specific RT-PCR on RNA from patient 4 revealed a minor product, indicating an abnormal transcript. **C**. Sanger sequencing of the RT-PCR product confirmed the absence of exon 9 in the patient 4. **D**. Diagram illustrating the effect of the c.826+5_826+9del on the mRNA splicing of FANCA. The deletion of exon 9 results in a premature stop codon in exon 10. **E**. Deletion/duplication analysis by a-CGH was additionally performed in the patient 4 and detected a heterozygous intragenic deletion of exons 21-28 in the *FANCA* gene.

**
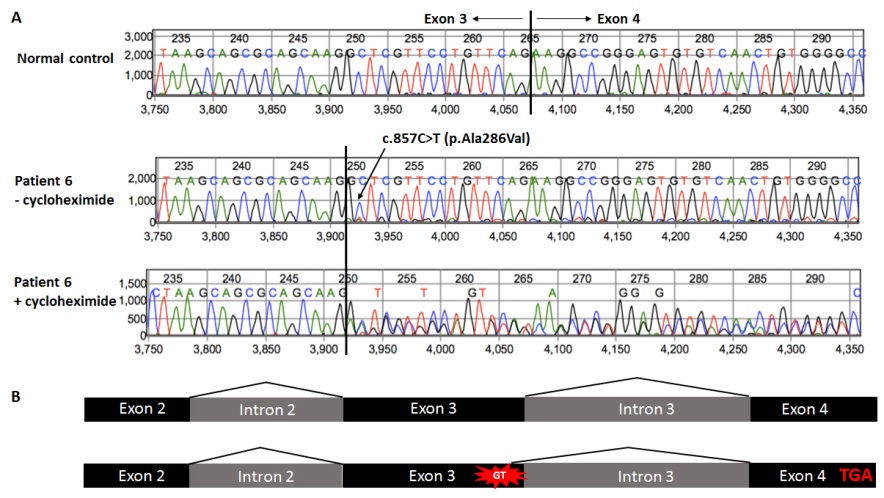
**

**Supplementary Figure 5**: Molecular characterization of the splicing effect of the c.857C>T variant in *GATA2* in the patient 6. **A**. Sanger sequencing of the RT-PCR product of RNA from the patient 6 skin fibroblasts untreated and treated with cycloheximide, and normal control. Patient 6 exhibits a 16 bp deletion in exon 3 which results in a premature stop codon in exon 4. **B**. Diagram of the effect of c.857C>T on the mRNA splicing in *GATA2*.

**Supplementary Table 1**: Panels and genes for hereditary MDS/AL predisposition testing.

| **Panels** | **Genes** |
| --- | --- |
| **Familial MDS/AL** | **ANKRD26**, **CEBPA**, DDX41, ETV6, **GATA2**, **RUNX1**, **SRP72**, **TERC**, **TERT**, **TP53**, **ATM**, **BLM** (**RECQL3**), **BRCA1**, **BRCA2**, **CBL**, **CHEK2** (**CHK2**), **MLH1**, **MSH2**, **MSH6**, **NBN** (**NBS1**), **NF1**, **PAX5**, **PMS2**, **PTPN11** |
| **IBMFS** | BRCA1 (FANCS), BRCA2 (FANCD1), BRIP1 (FANCJ),CSF3R, CTC1, DDX41, DKC1, ELANE (ELA2), ERCC4 (FANCQ), FANCA, FANCB, FANCC, FANCD2, FANCE, FANCF, FANCG, FANCI, FANCL, G6PC3, GATA2, GFI1, HAX1, MPL, NHP2, NOP10 (NOLA3), PALB2 (FANCN), RAD51 (FANCR), RAD51C (FANCO), RBM8A, RPL5, RPL11, RPL35A, RPS7, RPS10, RPS19, RPS24, RPS26, RTEL1, RUNX1, SBDS, SBF2, SLX4 (FANCP), SRP72, TERC, TERT, TINF2, UBE2T (FANCT), USB1 (C16orf57), VPS45, WRAP53 (TCAB1), WAS |
| **FA** | **BRCA1**(**FANCS**), **BRCA2** (**FANCD1**), **BRIP1** (**FANCJ**), **ERCC4** (**FANCQ**), **FANCA**, **FANCB**, **FANCC**, **FANCD2**, **FANCE, FANCF, FANCG, FANCI, FANCL**, **PALB2** (**FANCN**), **RAD51 (FANCR), RAD51C** (**FANCO**), UBE2T (FANCT), **SLX4** (**FANCP**), |
| **DBA** | **RPL5**, **RPL11**, **RPL35A**, **RPS7, RPS10,** **RPS19**, **RPS24**, **RPS26** |
| **DC/TBD** | **C16orf57**, **CTC1**, **DKC1**, **NOLA3** (**NOP10**), **NHP2**, **RTEL1**, **TERC**, **TERT**, **WRAP53 (TCAB1)**, **TINF2** |
| **SCN** | **CSF3R**, **ELANE** (**ELA2**), **G6PC3**, **GFI1**, **HAX1**, **VPS45**, **WAS** |

**Bold**: both sequencing and deletion/duplication analyzed

**Supplementary Table 2**: Variants selected for RNA splicing assay to clarify pathogenic nature.

| **Patient** | **Sample Type** | **Gene** | **Nucleotide** | **Zygosity** | ***In silico* Prediction^*^** | **Splicing assay result** | **Consistency of prediction with RNA splicing assay** |
| --- | --- | --- | --- | --- | --- | --- | --- |
| 4 | Blood | FANCA | c.826+5_826+9del | Het. | Interruption of  exon 9–intron9 junction | Exon 9 deletion | Yes |
| 6 | Skin fibroblasts | GATA2 | c.857C>T | Het. | Creation of de novo donor in exon 3 | 16-bp deletion in exon 3 | Yes |

Het., Heterozygote, **^*^**Alamut® Visual.

**REFERENCES**

1. Marchler-Bauer A, Bryant SH. CD-Search: protein domain annotations on the fly. *Nucleic Acids Res.* 2004;32(Web Server issue):W327-331.

2. Marchler-Bauer A, Anderson JB, Chitsaz F, et al. CDD: specific functional annotation with the Conserved Domain Database. *Nucleic Acids Res.* 2009;37(Database issue):D205-210.

3. Marchler-Bauer A, Lu S, Anderson JB, et al. CDD: a Conserved Domain Database for the functional annotation of proteins. *Nucleic Acids Res.* 2011;39(Database issue):D225-229.

4. Marchler-Bauer A, Derbyshire MK, Gonzales NR, et al. CDD: NCBI's conserved domain database. *Nucleic Acids Res.* 2015;43(Database issue):D222-226.

5. Folias A, Matkovic M, Bruun D, et al. BRCA1 interacts directly with the Fanconi anemia protein FANCA. *Hum Mol Genet.* 2002;11(21):2591-2597.

6. Ali AM, Pradhan A, Singh TR, et al. FAAP20: a novel ubiquitin-binding FA nuclear core-complex protein required for functional integrity of the FA-BRCA DNA repair pathway. *Blood.* 2012;119(14):3285-3294.

7. Collin M, Dickinson R, Bigley V. Haematopoietic and immune defects associated with GATA2 mutation. *Br J Haematol.* 2015;169(2):173-187.

8. Collins K. The biogenesis and regulation of telomerase holoenzymes. *Nat Rev Mol Cell Biol.* 2006;7(7):484-494.

9. Polprasert C, Schulze I, Sekeres MA, et al. Inherited and Somatic Defects in DDX41 in Myeloid Neoplasms. *Cancer Cell.* 2015;27(5):658-670.

10. Daly ME. Transcription factor defects causing platelet disorders. *Blood Rev.* 2016.
